# The Medical Voyage of Life

**Published:** 1883-10

**Authors:** 


### The Medical Voyage of Life.

The following clever chronological classification of the ills to which human flesh is heir, may give a faint conception of the gauntlet which we poor mortals have to run: First year: icterus neonatorum, hyperkinesis intestinalis and vaccination. Second year: dentition, croup, cholera infantum and fits. Third year: diphtheria, whooping cough and bronchitis. Fourth year: scarlatina, worms, and meningitis. Fifth year: measles. Now half the children are dead. Seventh year: mumps. Tenth year; chorea and typhoid fever. Fifteenth year: hyperæsthesia sexualis. Sixteenth year: spermatorrhœa, chlorosis and spinal irritation. Eighteenth year; bubo, alcoholic cephalalgia, vertigo. Twenty-fifth year: matrimony. Twenty-sixth year: insomnia de infanto. Thirtieth year: dyspepsia, nervous asthenia. Thirty-fifth year: pneumonia. Forty-fifth year: lumbago, presbyopia. Fifty-fifth year: rheumatism, alopecia. Sixtieth year: amnesia, deciduousness of teeth, bony arteries. Sixty-fifth year: apoplexy. Seventieth year: amblyopia, deafness, anosmia, general dyskinesis, atonic digestive tract, rheumatismus deformans. Seventy-fifth year: finis.—*The Medical Age.*

